# Complete Genome Sequence of *Cryobacterium arcticum* Strain PAMC 27867, Isolated from a Sedimentary Rock Sample in Northern Victoria Land, Antarctica

**DOI:** 10.1128/genomeA.00885-16

**Published:** 2016-09-01

**Authors:** Jaejin Lee, Ahnna Cho, Jae Young Yang, Jusun Woo, Hong Kum Lee, Soon Gyu Hong, Ok-Sun Kim

**Affiliations:** a Division of Polar Life Sciences, Korea Polar Research Institute, Incheon, Republic of Korea; b Division of Polar Earth-System Sciences, Korea Polar Research Institute, Incheon, Republic of Korea

## Abstract

*Cryobacterium arcticum* PAMC 27867, a psychrotolerant, Gram-positive bacterium, was isolated from a sedimentary rock sample collected at Eureka Spurs in northern Victoria Land, Antarctica. Here, we report the genome sequence of *C. arcticum* PAMC 27867.

## GENOME ANNOUNCEMENT

*Cryobacterium arcticum*, a psychrotolerant, Gram-positive, yellow-pigmented, aerobic, rod-shaped bacterium, was first isolated from Arctic soil (1). *C. arcticum* strain PAMC 27867 was isolated from a sedimentary rock sample (sandstone with exfoliated surface) collected at Eureka Spurs in northern Victoria Land, Antarctica (72°41′50″ S, 165°59′40″ E). Because mineral-weathering abilities are required to grow on rock surfaces, only a few microbes have been found in those habitats (2) and those microbes can be of great interest, especially in cold temperatures. Here, we report the genome sequence of *C. arcticum* PAMC 27867.

Genomic DNA of *C. arcticum* PAMC 27867 was extracted using the i-genomic BYF minikit (iNtRON Biotechnology, Republic of Korea) and used to construct a standard PacBio library with an average of 20 kb inserts. Genome sequencing of the strain PAMC 27867 was performed using Pacific Biosciences (PacBio) RS II single-molecule real-time (SMRT) sequencing technology (Pacific Biosciences, CA) (3). *De novo* assembly using the hierarchical genome assembly process (HGAP) pipeline in the SMRT analysis software version 2.3.0 was carried out for 49,687 reads with 10,280 nucleotides on the average (510,803,343 bp in total) (4), which resulted in one circular chromosome (112-fold coverage) and two circular plasmid (58-fold and 38-fold coverage) sequences. Protein coding sequences (CDSs) were predicted using Prokaryotic Dynamic Programming Genefinding Algorithm (Prodigal) version 2.6.1 (5). The functional annotation of predicted genes was conducted using UniProt (6), Pfam (7), COG (8), CAZy (9), and MEROPS (10) databases. The genome of *C. arcticum* PAMC 27867 consists of one circular chromosome of 4,174,501 bp and two plasmids (117,792 bp and 58,936 bp designated plasmid1 and plasmid2, respectively).

The annotation detected 2,855 CDSs including 10 rRNAs and 51 tRNAs. The annotated genome contains a gene encoding xanthorhodopsin and a gene cluster associated with biosynthesis of a carotenoid. The presence of these genes suggests that the strain could be a phototroph, which uses the bacterial rhodopsin with a carotenoid antenna. The strain PAMC 27867 may use diverse carbohydrates including cellulose, hemicellulose, levan, galactosidase, and mannose as carbon sources using glycosyl hydrolases such as endo-β-1,4-clucanase, cellobiohydrolase, endo-1,4-β-xylanase, levansucrase, and levanase. Genes encoding a methanol dehydrogenase and genes associated with ribulose monophosphate pathway for formaldehyde assimilation were also found. There are two genes encoding low temperature requirement proteins and four genes encoding cold shock proteins. The genome possesses genes encoding nitrate reductase subunits, nitrite reductase subunits, and glutamine synthetases. Because all pathways for biosynthesis of amino acids are present in the genome, it may imply that PAMC 27867 plays an important role as a supplier of amino acids in habitats. Plasmids encode many putative transmembrane proteins, some of which are related to metal transport systems (e.g., a cadmium transporter). In addition, plasmids encode proteins related to detoxification of metal such as copper oxidase and alkylmercury lyase.

### Accession number(s).

The genome sequence of *Cryobacterium arcticum* PAMC 27867 has been deposited at GenBank under the accession numbers CP016282 (chromosome), CP016283 (plasmid1), and CP016284 (plasmid2). The strain PAMC 27867 is available from the Polar and Alpine Microbial Collection (Korea Polar Research Institute, Incheon, Republic of Korea).
